# Training health care providers to administer VIA as a screening test for cervical cancer: a systematic review of essential training components

**DOI:** 10.1186/s12909-023-04711-5

**Published:** 2023-09-28

**Authors:** Thea Beate Brevik, Lara Rodrigues da Matta Calegari, Isabel Mosquera Metcalfe, Petter Laake, Mauricio Maza, Partha Basu, Adam Todd, Andre L. Carvalho

**Affiliations:** 1 Faculty of Health Sciences and Social Care, Molde University College, Molde, Norway; 2 Clinic of Surgery, Møre and Romsdal Hospital Trust, Molde Hospital, Molde, Norway; 3 Mackenzie Evangelical School of Medicine, Curitiba, Paraná Brazil; 4 Early Detection, Prevention, and Infections Branch, International Agency for Research on Cancer, Lyon, France; 5 Oslo Centre for Biostatistics and Epidemiology, Department of Biostatistics, University of Oslo, Oslo, Norway; 6 Department of Noncommunicable Diseases and Mental Health, Unit of Noncommunicable Diseases, Violence, and Injury Prevention, Pan American Health Organization, Washington, DC USA; 7 School of Pharmacy, Newcastle University, Newcastle upon Tyne, UK; 8 Centre for Global Health Inequalities Research (CHAIN), Norwegian University of Science and Technology (NTNU), Trondheim, Norway

**Keywords:** Education, cervical cancer, Cancer prevention, Screening, Training, Women’s health, global health, systematic review

## Abstract

**Background:**

Training health care providers to administer visual inspection after application of acetic acid (VIA) is paramount in improving cervical cancer screening services for women in low- and middle-income countries. The objective of this systematic review was to create a framework of essential VIA training components and provide illustrating examples of how VIA training programs can be carried out in different clinical settings.

**Methods:**

A systematic review of PubMed, Embase, and Web of Science (from 2006 to 2021) was undertaken. Our inclusion criteria comprised articles reporting on implemented cervical cancer screening programs using VIA in a screen-and-treat approach. Trained health care providers with any level of health education were included, and the outcome of interest was the reporting of training components. Data were extracted by two reviewers, and a narrative synthesis of the training programs was performed. We developed a framework of seven essential training components and applied it to assess how training courses were conducted in different settings.

**Results:**

13 primary studies were eligible for inclusion, including 2,722 trained health care providers and 342,889 screened women. Most training courses lasted 5–7 days and included theoretical education, practical skill development, and competence assessment. It was unclear how visual aids and training in client counselling and quality assessment were integrated in the training courses. After the training course, nearly all the VIA training programs made provisions for on-job training at the providers’ own clinical settings through supervision, feedback, and refresher training.

**Conclusions:**

This study demonstrates the feasibility of implementing international training recommendations for cervical cancer screening in real-world settings and provides valuable examples of training program implementation across various clinical settings. The diverse reporting practices of quality indicators in different studies hinder the establishment of direct links between these data and training program effectiveness. To enhance future reporting, authors should emphasize specific training components, delivery methods, and contextual factors. Standardized reporting of quality indicators for effective evaluation of VIA training programs is recommended, fostering comparability, facilitating research, and enhancing reporting quality in this field.

**Supplementary Information:**

The online version contains supplementary material available at 10.1186/s12909-023-04711-5.

## Introduction

Cervical cancer ranks as the fourth most frequently diagnosed cancer and the fourth leading cause of cancer death in women, with an annual estimated 604,127 cases and 341,831 deaths worldwide [1]. A disproportionate number occur among women living in low-and-middle-income countries (LMICs) [2]. The World Health Organization (WHO) recommended in their guidelines published in 2013 visual inspection after application of acetic acid (VIA) as the most feasible and affordable alternative to cytology screening for the LMICs [3]. VIA involves naked-eye examination of the uterine cervix with appropriate illumination after application of 3–5% acetic acid solution [3]. The test aims to detect precursor lesions as well as early cervical cancers in asymptomatic women [3]. VIA is widely used as a screening test in LMICs, often in a ‘screen and treat’ approach where screen-positive women are offered immediate treatment [4]. Such an approach has been demonstrated to reduce the number of clinic visits by women, improve compliance with treatment, and make the program efficient [4].

The paradigm of cervical cancer screening is evolving rapidly [4]. WHO updated their guideline on cervical cancer screening in July 2021 [5], which recommends Human Papillomavirus (HPV) DNA detection as the gold standard for primary testing for cervical cancer screening, rather than VIA or cytology in a ‘screen and treat’ approach for women in the general population. For women living with Human Immunodeficiency Virus (HIV), a ‘screen, triage and treat’ approach is recommended when using HPV detection [5]. Despite these recommendations, many countries with limited resources will have to continue with VIA as the primary screening test till they have enough resources to introduce HPV detection tests [4]. In the HPV screen and treat algorithm, health care providers will still need similar clinical training to visually triage women eligible for treatment. In some countries, VIA will have a key role as a triage test even after introduction of HPV test, especially in countries with high HIV prevalence [4].

VIA can generally be performed by health care providers after a short period of training [3]. The interpretation of the test is based on the detection of a well-defined opaque acetowhite area on the transformation zone of the cervix appearing one minute after the application of acetic acid solution [3]. Studies have found that the provider’s professional background (e.g., physicians, nurses, health workers) does not influence the test accuracy of VIA [6], and that trained non-physicians can perform VIA screening while maintaining high-quality services [3, 7]. However, due to the subjective nature of the test, success of the screening program depends on the high-quality training of providers [4]. Worldwide, there are many training manuals providing guidelines on VIA training [7–14]. However, at present, it is unknown to what extent the providers of VIA are trained or how far the guidelines on training are adhered to. Designing an effective training program can be a complex process, and although most of the principles, steps, and interpretations remain similar, the contexts and settings may differ. The objective of this systematic review was to create a framework of essential VIA training components and provide illustrating examples of how VIA training programs can be carried out in different clinical settings.

## Methods

The review protocol is registered with Prospero (CRD42021220497). The review process and reporting were guided by the Preferred Reporting Items for Systematic Reviews and Meta-Analyses (PRISMA) guidelines [15] (Table S1).

### Eligibility criteria

We included studies reporting on implemented cervical cancer screening programs using VIA in a screen-and-treat approach. Our inclusion criteria comprised articles reporting on programs implemented after the year 2005. This specific timeframe was chosen because it aligns with a landmark publication in 2005, which presented essential training components in VIA screening, representing an international consensus among members of the Alliance for Cervical Cancer Prevention (ACCP) [8]. The participants (trainees) could have any level of health education, and the outcome of interest was the reporting of the VIA training components. We included studies published in English in peer-reviewed journals, and excluded studies only available as abstracts.

### Literature search

Searches were conducted in PubMed, Embase, and Web of Science; the initial search was undertaken in December 2020 and updated in October 2021 (Table S2). We searched for papers published after 2005. PICO forms (Population, Intervention, Control, and Outcome) were used to create a structured and precise search strategy (Table S3). The search strategy and history were critically assessed using the evidence-based checklist of peer review of electronic search strategies [16]. The reference lists of included articles were also hand searched for further relevant articles.

### Study selection

The study assessment was conducted in Covidence by two reviewers (TB and LC). First, TB and LC independently reviewed a randomly selected 10% of the titles and abstracts. The interrater reliability (IRR) was measured using Cohen’s Kappa coefficient (κ). It was predetermined that the two reviewers would individually screen abstracts until they reached a κ > 0.7. After reaching this agreement, TB continued with the title and abstract assessment. The full text assessment was conducted independently by both TB and LC. Any discrepancies between the two reviewers were resolved through discussion. If agreement could not be reached, the project leader (AC) was consulted who had the final say in the decision. Excluded abstracts and articles were categorized in Covidence and made available to the review team to ensure transparency throughout the process.

### Data extraction

Information was extracted for each study using a standardized form, which included the following variables: the contextual settings (country, healthcare setting, and year of VIA implementation), target population eligible for screening, trainees (number and professions), information relating to the training program, and results (timeframe of follow-up, screening participation rates, and VIA positivity (+) rates). Data extraction was carried out by TB and LC. Disagreements were resolved by discussion, and if agreement could not be reached, the project leader (AC) had the final say.

### Framework to conceptualize VIA training

Informed by the Alliance for Cervical Cancer Prevention (ACCP) [8], and after discussion with screening experts at the International Agency for Research on Cancer (IARC), a framework to conceptualize essential VIA training components was developed (Table 1).

**Table 1:** Description of essential training components

Essential training components	Description
1) Training course delivered over a defined period of time	The length of the training course should depend on the trainees’ skill level at baseline and the amount of clinical practice available during training. The training should be long enough to ensure that the VIA screening services are delivered with both competence and confidence. The training should take place in a real clinical setting, if not the actual service-delivery site. A 5 to 10- day duration of training course is generally considered as appropriate for the trainees (clinicians, nurses, and midwives) to obtain adequate knowledge and clinical skills to deliver services competently. In a real health service setting it is challenging for the health professionals to leave their routine job for a longer duration to attend such targeted training.
2) Theory-based education	The training course should contain theory-based elements that cover the fundamental purpose, principles, and the specifics of the VIA procedure. There should be an emphasis on anatomy, physiology, and the etiology of cervical cancer at a level that is suitable for the selected trainees and that is highly practical. Understanding how VIA is performed and the interpreting the test by the nature of acetowhite reaction is required.
3) Hands-on competency-based skill acquisition	The training course should include practical hands-on experience that ensures that each trainee can practice the VIA technique on an adequate number of women and, ideally, should be exposed to both test-positive and test-negative women.
4) Client counselling	Trainees should be trained to counsel women about the VIA screening process. Trainees should also know how to counsel a woman who is VIA-positive or who has cervical cancer, including the risks and benefits of the treatment methods offered. Training in counselling can take many forms, like watching video or real-life demonstrations, practicing in a group, or counselling a client as part of the VIA procedure.
5) Visual aids	The training course should contain visual aids to show trainees the spectrum of cervical diseases and normal physiological changes that may be observed. Photographs, digital images, flash cards, and interactive CD-ROMs are valuable supplements to the learning process. Images should be in color and accompanied with VIA diagnosis from an expert for real-time comparison.
6) Competency assessment	At the end of the training course, the trainees should demonstrate the performance of all the steps of a procedure correctly and in the right order without prompting from a trainer. The trainee’s competency is best assessed with a performance checklist, and a specific score can be required as part of the successful completion of a training course.
7) Quality assurance	The training course should incorporate a quality-assurance module into the general training to allow the trainees to understand the philosophy of quality assurance, its necessity and required components, and how quality assurance will affect their overall performance. The depth of information presented may vary, but the overall value of quality assurance and how to train people in quality assurance are core concepts. Supplying information about quality assurance relates to the way(s) in which records are kept, information is documented, and programs are tracked. Teaching providers to be effective supervisors is another required element of quality-assurance training.

A table illustrating the seven key components of VIA training programs

### Data synthesis

When synthesizing the reported information on the VIA training, one reviewer (TB) carried out a dichotomy coding (Yes/No) for the presence of the seven components in each of the included studies. LC checked the coding, while AC was consulted for consensus on disagreements. Since many authors did not describe their interventions in detail probably due to word limitations of publications, we contacted all the corresponding authors to obtain more information about their training intervention. Out of 13 corresponding authors contacted, nine responded and provided additional information about the VIA training.

## Results

### Search results

**Fig. 1:**
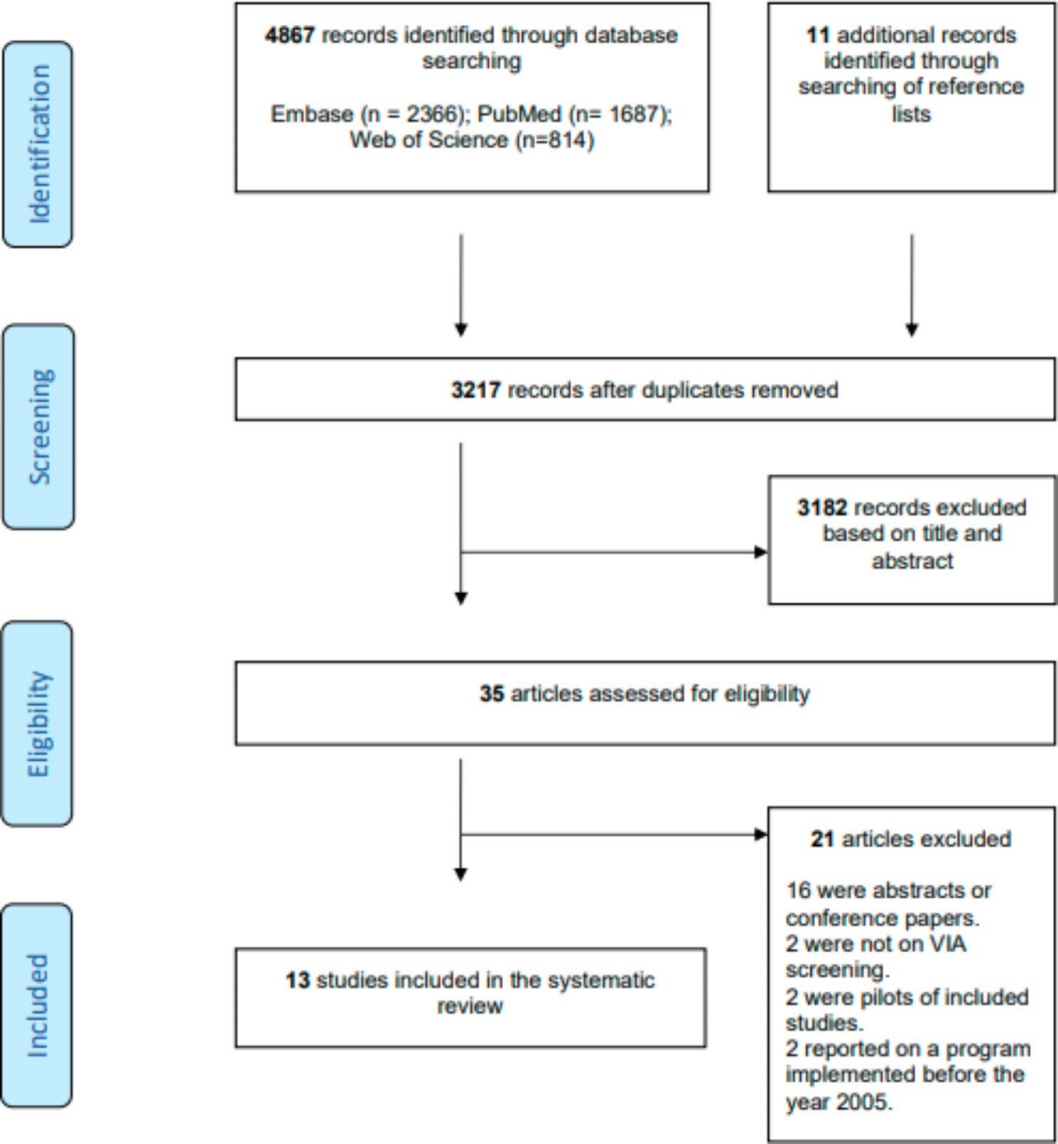
Flow diagram. Prisma flow chart of search results and study selection

The database searches identified 4867 records: after title and abstract screening, 35 full papers were assessed for eligibility (Fig. 1). In total, 13 primary studies were included in this systematic review [16–29].

### Characteristics of included studies

The included studies reported on implemented screen-and-treat programs in Botswana [27], Burkina Faso [19], Cameroon [26], Eswatini [28], Ethiopia [20], Guyana [18], India [21], Indonesia [17], Malawi [22], Nigeria [23], Tanzania [24, 29], and Zambia [25]. The programs were delivered in community- or hospital-based clinics, with many using existing health facilities, such as HIV clinics. The years the training programs were conducted were between 2006 [25] and 2017 [22]. Four studies [18, 20, 22, 27] specifically targeted HIV positive women in their screening approach. In total, the studies included in the final analysis reported to have screened 342,889 women, ranging from 556 women screened in 5 days [24] to 102,942 women screened in 7 years [25]. The total number of VIA trainees was 2,722 providers, ranging from 3 [28] to 2216 [17], although we note two studies [25, 27] did not report the number of trainees. The trainees included nurses [17–20, 22–29], midwives [16–20, 23, 27, 28], physicians [16–21, 23, 29], and other health care workers with less formal health education [18, 19, 24, 25, 30]. More information about the included studies is available in Table 2.

**Table 2:** Characteristics of included studies

Included article	Years of program implemented	Country implemented	Age-specific inclusion criteria	Number of women screened	Average VIA + rate over the length of the program	Number of trainees trained	Health education level of the trainees included in the training program
Nuranna et al., 2012 (17)	2007–2010	Indonesia	Unknown.	22,989	4,2% 970/ 22,989) over 3 years.	2216	General practitioners, midwifes, public health cadres, and key people from the society.
Martin et al., 2014 (18)	2009–2012	Guyana	25–49 years.	21,597	13% (2860/ 21,597) over 42 months.	71	Physicians, nurses, midwifes, and medical examiner.
Ouedraogo et al., 2018 (19)	2010–2014	Burkina Faso	25–59 years.	13,999	8,9% over 4 years.	60	Gynecologists, general practitioners, and nurse-midwifes.
Shiferaw et al. 2016 (20)	2010–2014	Ethiopia	30–45 years.	16,527	10% (1656/ 16,527) over 4 years.	77	Nurses, midwifes, and physicians.
Shikha et al., 2020 (21)	2014–2017	India	30–60 years.	100,836	5,4% (5477/ 100,863) over 3 years.	150	Obstetricians, gynecologists, and general practitioners.
Talama et al., 2020 (22)	2017–2018	Malawi	25–49 years.	547	3,9% over 1 year.	6	Nurses.
Awolude, Oyerinde et Akinyemi, 2018 (23)	2016–2017	Nigeria	All women.	950	6,9% (66/ 950) over 1 year.	51	Physicians, nurses, midwifes, and community health workers.
Bernstein et al., 2018 (24)	2016	Tanzania	All women.	556	10,6% (59/ 556) over 5 days.	11	Health care workers.
Parham et al., 2015 (25)	2006–2013	Zambia	All women.	102,942	20% (20,319/ 101,867) over 7 years.	Unknown.	Nurses.
DeGregorio et al., 2017 (26)	2007–2014	Cameroon	HIV-positive women > 21 years, and HIV-negative women or unknown status > 25 years.	44,979	9% (4042/ 44,979) over 8 years.	25	Nurses.
Ramogola-Masire et al., 2012 (27)	2009–2011	Botswana	All women.	2175	11,6–35% (253 + 506/ 2175) over 2 years.	Unknown.	Nurses and midwifes.
Asgary et al., 2020 (28)	2016–2018	Eswatini	25–49 years.	4247	13,4% (570 /4247) over 1,5 years.	3	Nurses and midwifes.
Yeates et al., 2020 (290)	2016–2017	Tanzania	> 25 years.	10,545	Unknown.	52	Nurses, clinical officers, assistant medical officers, and obstetricians/gynecologists.

### VIA training courses

The VIA training courses were heterogeneous with substantial variability in their objectives, structure, content, duration, and reporting. In all studies, VIA was implemented in a screen-and-treat approach, where 5 studies [24–29] integrated digital technologies to enhance performance of VIA, such as using digital imaging [27] or smartphones [29]. Table 3 outlines the reported training components in each of the included studies.

**Table 3:** The reporting of essential components for VIA training courses

Included article (ref)	Year of implementation	Screening program approach	Duration of training course	Theoretical education	Practical hands-on	Client counselling	Visual aids	Competency assessment	Quality assurance
17	2007	Screen-and-treat (S&T)	5 days	Y	Y			Y	
18 *	2009	S&T	6 days	Y	Y	Y*	Y*	Y*	Y*
19 *	2010	S&T	6 days	Y	Y	Y	Y	Y*	Y
20 *	2010	S&T	5 / 10 days	Y	Y	Y	Y	Y*	Y*
21 *	2014	S&T	3 days	Y	Y	Y*	Y*	Y*	
22 *	2017	S&T	5 days	Y	Y*			Y*	
23	2016	S&T	5 days	Y	Y	Y	Y	Y	Y
24*	2016*	S&T	5 days	Y	Y		Y	Y	
25	2006	Digital enhanced S&T	2 weeks	Y	Y				
26 *	2007	Digital enhanced S&T	2 weeks	Y	Y*	Y*	Y	Y	Y*
27	2009	Digital enhanced S&T	8,5 weeks	Y	Y			Y	Y
28 *	2016	Digital enhanced S&T	1 week	Y	Y		Y	Y*	
29 *	2016	Digital enhanced S&T	6 days	Y*	Y*		Y*	Y	

* Additional information retrieved through email correspondence with corresponding author

Most training programs were based on international training guidelines, such as the WHO’s guide to essential practice in comprehensive cervical cancer control [12, 13] and adapted to the specific setting. One study [27], undertaken in Botswana, trained nurses because they were more available than physicians and familiar with performing pelvic exams. Another study [21] trained private practitioners in India, based on the long-standing association of working with doctors in public health programs. A third study [23] trained community health workers in Nigeria because nurses and doctors were largely absent in rural communities.

### Training duration

Most of the VIA training courses lasted between 5 and 7 days. In one study [20], for Ethiopian nurses and midwives, the training course lasted for 10 days, while for obstetricians and gynecologists, it was 5 days. In contrast, one study [17] implemented a standardized 5-day training course for over 2000 trainees in Indonesia: the same training course was provided to doctors as well as community health workers. Three studies [24–27] organized training courses that lasted between 2 and 8 weeks to train the trainees in both VIA screening and new digital technology. Another study [29] included providers who had previously completed a 6-days training course and screened more than 50 women, before organizing a new training program focusing on technical training on smartphone-enhanced VIA.

### Theoretical training

All studies reported a theoretical educational component in the training program. In general, however, the included studies reported limited information about the content of the theoretical sessions. The main areas covered were education on: female genital anatomy and cervical cancer pathophysiology; the VIA screening procedure; recognition and interpretation of features on VIA; appropriate treatment and referral of VIA positive women; and, infection prevention. Some of the training courses also included information about specific considerations, such as characteristics of cervical cancer in HIV positive women. One study [18] chose to arrange adaptive educational sessions that focused on the trainees’ weaknesses identified through an initial assessment of baseline knowledge and skills.

### Practical hands-on

All the reported training courses included practical hands-on sessions, either classroom-based or clinic-based, although the sessions were conducted in different ways. In one study [17], the trainees went through a one-day live demonstration of VIA, after which they were able to practice on women in a clinic under supervision of appropriately trained professionals. Another study [24] reported that trainees had four days of hands-on training with women called from the community to undergo VIA. A third study [23] reported that trainees were trained in the classroom on techniques related to insertion of a vaginal speculum (with identification of cervix using a pelvic model), visual inspection of the cervix, test sampling, and application of acetic acid (simulated learning).

### Client counselling

Overall, the included studies contained limited information regarding how trainees were trained in client counselling. One study [22] reported that the lecture topics included counselling and informed choice, while the classroom-based practical sessions covered post-test counselling of patients. Another study [19] used checklists to validate the trainees’ skills in interpersonal communication and counselling. Although the studies reported limited information on the training of client counselling, many of the included studies emphasized that participating women were counselled in the clinical setting about the screening techniques and any side effects that may arise. One study [24] described that healthcare staff explained to the women in Swahili the risks and benefits of the VIA screening procedure, including the meaning and consequences of a positive test and the availability of treatment. One study [27] highlighted that the technology produced high resolution images that could be used to educate and counsel the women, and this was included as a component of the training program.

### Visual aids

Some of the studies reported the use of visual aids to support training, such as photographic images [19, 23, 24], flash cards [20, 28], educational videos [20], and PowerPoint presentations [28]. One study [24] reported that several de-identified patient cervical images of VIA-positive and -negative examples were shown to ensure that the trainees could practice categorizing clinical impression. Two studies [19, 23] reported using anatomic models for identification of the cervix.

### Competency assessment

Several studies reported that the trainees’ competence was assessed at the end of the training course. In one study [17], each trainee had to perform VIA on 100 women, out of which the 2–3 VIA positive cases were confirmed by the supervisor. Another study [29] considered the trainees as graduated from the training program based on three key factors: the number of women screened, the level of agreement with reviewers, and meeting a specific threshold for the number of VIA positive women. In one study [27], each trainee had to successfully perform 100 VIA examinations, 100 digital photographs, and 35 cryotherapies.

### Quality assurance

Few studies reported on including a quality-assurance module into the general training. One study [23] reported that the educational lectures covered topics on recording, appropriate documentation, and referral systems. Another study [19] let the trained providers collect and monitor the data together with the researchers, which allowed the providers to visualize progress, analyze trends, evaluate themselves, and identify potential bottlenecks in service provision. Furthermore, this approach empowered the trained providers to track progress, identify gaps, and take corrective actions to remedy any shortcomings, thereby reaching more successful outcomes [19].

### Continued training in VIA

After the training course, nearly all the VIA training programs made provisions for on-job training at the providers’ own clinical settings through supervision, feedback, and refresher training. On-job supervision was provided by allowing the trained providers to work in pairs with experienced gynecologists or nurses who supervised their practice. Regular supportive supervision visits from experts were provided to offer transfer of learning. In one study [25], nurses visited rural facilities every three months for purposes of quality assurance and continued medical education. On-job feedback and mentoring were provided by experts reviewing the cervical images, captured during VIA by the providers, on a regular basis. Such regular meeting with experts offered feedback and education [28], increased understanding [27], and arrival at consensus opinions for treatment options [26]. Regularly organized refresher training and workshops were also used to retain or enhance VIA competency.

**Fig. 2:**
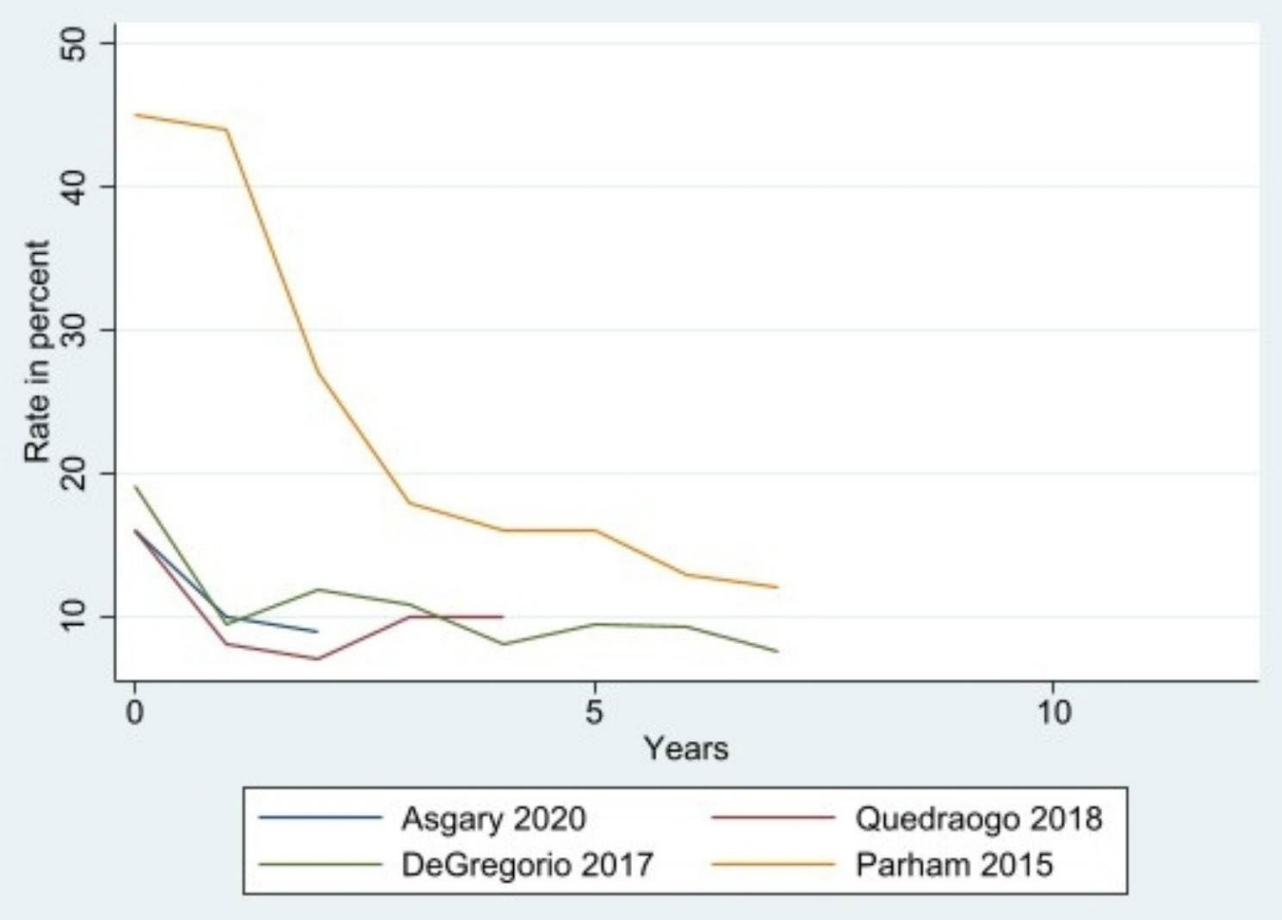
VIA positivity rates over time. Four studies reported serial point estimates of the VIA + rate in percentage over years. The four studies all provided prolonged training after the initial training course and showed that the VIA + rates reached the expected level over time. The VIA + in general population of women aged 30–60 years normally ranges between 5 and 10%

Needs for additional training or mentorship of the providers were identified through measuring the VIA + rates as a performance quality indicator for monitoring and evaluation purposes. Studies have shown that the VIA + rate in general population of women aged 30–60 years ranges between 5 and 10% [31]. The positivity rates tend to be higher in settings with a higher HIV prevalence. If the test positivity is too low, there is a possibility of missing the disease, while if it is too high, there is a higher possibility of false positives [31]. However, it is more important to measure VIA + serially over time to check if the rate is stable. In one study [28], the VIA + rate was at 16% after the initial training but increased to 40% after nine months. After refresher training and continued mentoring were implemented, the positivity rate decreased to an average of 6.3%, which was maintained all along the program [28]. In another study, the proportion of women considered to have inadequate VIA test reduced following additional training of nurses to better expose the endocervical canal [26]. Although all the included studies reported the average VIA + rate over the length of their screening program, only four studies [19, 25, 26, 28] reported serial point estimates of the VIA + rate over time (Fig. 2). These four studies all provided prolonged training after the initial training course and showed that the VIA + rates reached the expected level over time.

## Discussion

In this systematic review, we developed a framework to conceptualize essential VIA training components and have applied that framework to the literature to determine how extensive VIA training has been carried out in different settings. Our findings indicate that implemented VIA training programs have been carried out in line with international recommendations [7–11], but more importantly, that the training recommendations are feasible to implement in real settings. This observation holds paramount importance as previous studies have shown that with good training and sustained quality assurance and monitoring, screening of women with VIA followed by appropriate management of screen-positive women can reduce cervical cancer incidence and mortality [4, 32]. However, VIA performance varies widely, and the VIA + rates have shown high variability between countries [33] and within the same program or setting [34]. The sensitivity reported from some real programmatic settings ranged from 25 to 82% [33, 35]. Inconsistency across studies reflects the substantial subjectivity in interpreting visual tests by different providers, due to different levels of competencies, training methods, monitoring and quality assurance, and the low reproducibility of visual inspection methods [33]. Notably, the accuracy of VIA has been found to increase significantly by study phase [33], emphasizing the importance of experience, continuous training, and supervision.

A major pitfall in training is the lack of specific recommendation from international organizations. One size may not fit all, but some training guidance on the minimum requirements (e.g., duration of training, number of cases to be observed, and trainees-to-trainer ratio) will be very helpful. As the new WHO guidelines [5] refer to the screen-and-treat approach based on HPV primary screening followed by visual triage for treatment, it is important to have clear standards on how training should be conducted. Health care providers must be trained to visually triage women eligible for ablative treatment (cryotherapy or thermo-coagulation) based on their HPV status and not on the presence of acetowhite lesions. Still, VIA will have a key role as a triage test after introduction of HPV test in some countries. A high proportion of women with a positive HPV test will not necessarily have cervical precancer or cancer, and to reduce the referral for all HPV-positive women for colposcopy and/or treatment, many countries will use VIA to triage HPV-positive women [4]. In setting with high prevalence of HIV, health care providers will continue to perform VIA to triage women. WHO Academy is collaborating with IARC to develop a comprehensive learning program for providers of cervical cancer screening and treatment.

We found that many of the included studies reported that their training program was adapted to their specific settings, but without explaining which kind of adaptations were done and on what basis. Without a complete published description regarding the details of the training programs, it can be challenging to implement courses that are known to be successful and replicate or build on the research findings [36]. In a systematic review on the context in which cervical cancer screening is delivered in India, the authors mention that many of the included studies did not provide any information on the training [37]. To improve future reporting on training to support cancer screening, we encourage authors to highlight the specific components of the training program, including how the training was delivered, and in what context. There are similar reporting tools in the literature, which could be adapted for this purpose; for example, the TIDieR (Template for Intervention Description and Replication) checklist [36] that is used to report interventions in healthcare.

Initially, our aim was to explore the success of each training program by analyzing quality performance indicators, such as VIA + rates, treatment, and referral rates. However, the quality performance indicators were too diversely collected and reported between studies to link these data to the effectiveness of training programs. We found indications that VIA + rates can reach an acceptable level over time when prolonged training is provided. However, this finding is based on data from only four studies, which emphasizes the need for more data. Monitoring and evaluation of services are required, and we recommend that quality performance indicators are collected and monitored regularly. One proposition on the collection of data is the CanScreen5 – Cervical Cancer Screen-and-treat Quantitative Data Collection Form [38], which focuses on the target population, screening test outcomes, further assessment outcomes, cancer staging, and treatment.

For future reviewers to be able to explore the effectiveness of VIA training programs, we recommend a more homogeneous reporting of the quality indicators. We recommend that, at the very least, studies report VIA + rates measured after training along with corresponding point estimates over time. We propose that articles on VIA training include a table containing monthly or quarterly data (depending on the study duration) on the following quality indicators as a minimum: Number of women screened using VIA, VIA + rate, same-day treatment rates (cryotherapy or thermo-coagulation), and referral rates for excision procedures. This approach will provide a more comprehensive and standardized representation of the study findings, facilitating comparisons, and improving the overall quality of reporting in the research field.

### Study limitations

Our search strategy was designed to identify studies on provider-directed interventions on cancer screening participation among disadvantaged populations, which led to some relevant keywords, such as “VIA”, being missing. Nevertheless, the search includes relevant keywords related to cervical cancer screening, provider training, and screening participation. We also conducted manual searches to identify additional papers of interest, ensuring a comprehensive approach. Additionally, we acknowledge that our restriction to studies published in English may have led to the oversight of relevant studies, particularly in regions where English is not the primary language.

## Conclusion

This study demonstrates the feasibility of implementing international training recommendations for cervical cancer screening in real-world settings and provides valuable examples of training program implementation across various clinical settings. However, the diverse reporting practices of quality indicators in different studies hinder the establishment of direct links between these data and training program effectiveness. To enhance future reporting on training programs supporting cancer screening, we encourage authors to emphasize specific training components, delivery methods, and contextual factors. For effective evaluation of VIA training programs, we recommend a more standardized reporting of quality indicators. At the very least, studies should report VIA + rates measured after training, along with corresponding point estimates over time. Additionally, we suggest the reporting of monthly or quarterly data for essential quality indicators. Adopting these practices will improve comparability, facilitate research, and enhance the overall quality of reporting in this field.

### Electronic supplementary material

Below is the link to the electronic supplementary material.

Supplementary Material 1

## Data Availability

The datasets used and/or analyzed during the current study are available from the corresponding author on reasonable request.

## Abbreviations

ACCP: the Alliance for Cervical Cancer Prevention
HIV: Human Immunodeficiency Virus
HPV: Human Papillomavirus
IARC: the International Agency for Research on Cancer
IRR: Interrater Reliability
κ: Cohen’s Kappa coefficient
LMICs: Low- and Middle- Income Countries
PICO: Population, Intervention, Control, and Outcome
PRISMA: Preferred Reporting Items for Systematic Reviews and Meta-Analyses
VIA: Visual Inspection after application of acetic acid
VIA+: VIA positivity
WHO: The World Health Organization
