# Supplementary material for: Training health care providers to administer VIA as a screening test for cervical cancer: a systematic review of essential training components
Source: BMC Med Educ. 2023 Sep 28;23:712. doi: 10.1186/s12909-023-04711-5 (PMC10540456; doi:10.1186/s12909-023-04711-5)
Supplement: Supplementary file 1 — Supplementary Material 1 [file 12909_2023_4711_MOESM1_ESM.docx]

**SUPPLEMENTAL TABLE 1: PRISMA checklist**

| **Section and Topic** | **Item #** | **Checklist item** | **Location where item is reported** |
| --- | --- | --- | --- |
| **TITLE** | | |  |
| Title | 1 | Identify the report as a systematic review. | p. 1 |
| **ABSTRACT** | | |  |
| Abstract | 2 | See the PRISMA 2020 for Abstracts checklist. | p.1-2 |
| **INTRODUCTION** | | |  |
| Rationale | 3 | Describe the rationale for the review in the context of existing knowledge. | p. 3-4 |
| Objectives | 4 | Provide an explicit statement of the objective(s) or question(s) the review addresses. | p. 4 |
| **METHODS** | | |  |
| Eligibility criteria | 5 | Specify the inclusion and exclusion criteria for the review and how studies were grouped for the syntheses. | p. 5 |
| Information sources | 6 | Specify all databases, registers, websites, organisations, reference lists and other sources searched or consulted to identify studies. Specify the date when each source was last searched or consulted. | p. 5 |
| Search strategy | 7 | Present the full search strategies for all databases, registers and websites, including any filters and limits used. | Supplemental file 2 and 3 |
| Selection process | 8 | Specify the methods used to decide whether a study met the inclusion criteria of the review, including how many reviewers screened each record and each report retrieved, whether they worked independently, and if applicable, details of automation tools used in the process. | p. 5-6 |
| Data collection process | 9 | Specify the methods used to collect data from reports, including how many reviewers collected data from each report, whether they worked independently, any processes for obtaining or confirming data from study investigators, and if applicable, details of automation tools used in the process. | p. 6 |
| Data items | 10a | List and define all outcomes for which data were sought. Specify whether all results that were compatible with each outcome domain in each study were sought (e.g. for all measures, time points, analyses), and if not, the methods used to decide which results to collect. | p. 6  p. 25-26 (T1) |
|  | 10b | List and define all other variables for which data were sought (e.g. participant and intervention characteristics, funding sources). Describe any assumptions made about any missing or unclear information. | p. 6 |
| Study risk of bias assessment | 11 | Specify the methods used to assess risk of bias in the included studies, including details of the tool(s) used, how many reviewers assessed each study and whether they worked independently, and if applicable, details of automation tools used in the process. | - |
| Effect measures | 12 | Specify for each outcome the effect measure(s) (e.g. risk ratio, mean difference) used in the synthesis or presentation of results. | - |
| Synthesis methods | 13a | Describe the processes used to decide which studies were eligible for each synthesis (e.g. tabulating the study intervention characteristics and comparing against the planned groups for each synthesis (item #5)). | p. 6 |
|  | 13b | Describe any methods required to prepare the data for presentation or synthesis, such as handling of missing summary statistics, or data conversions. | p. 6 |
|  | 13c | Describe any methods used to tabulate or visually display results of individual studies and syntheses. | p. 7  F2 |
|  | 13d | Describe any methods used to synthesize results and provide a rationale for the choice(s). If meta-analysis was performed, describe the model(s), method(s) to identify the presence and extent of statistical heterogeneity, and software package(s) used. | - |
|  | 13e | Describe any methods used to explore possible causes of heterogeneity among study results (e.g. subgroup analysis, meta-regression). | - |
|  | 13f | Describe any sensitivity analyses conducted to assess robustness of the synthesized results. | - |
| Reporting bias assessment | 14 | Describe any methods used to assess risk of bias due to missing results in a synthesis (arising from reporting biases). | - |
| Certainty assessment | 15 | Describe any methods used to assess certainty (or confidence) in the body of evidence for an outcome. | - |
| **RESULTS** | | |  |
| Study selection | 16a | Describe the results of the search and selection process, from the number of records identified in the search to the number of studies included in the review, ideally using a flow diagram. | p. 7  p. 31 (F1) |
|  | 16b | Cite studies that might appear to meet the inclusion criteria, but which were excluded, and explain why they were excluded. | p. 31 (F1) |
| Study characteristics | 17 | Cite each included study and present its characteristics. | p. 7  p. 27-29 (T2) |
| Risk of bias in studies | 18 | Present assessments of risk of bias for each included study. | - |
| Results of individual studies | 19 | For all outcomes, present, for each study: (a) summary statistics for each group (where appropriate) and (b) an effect estimate and its precision (e.g. confidence/credible interval), ideally using structured tables or plots. | p. 30 (T3) |
| Results of syntheses | 20a | For each synthesis, briefly summarise the characteristics and risk of bias among contributing studies. | - |
|  | 20b | Present results of all statistical syntheses conducted. If meta-analysis was done, present for each the summary estimate and its precision (e.g. confidence/credible interval) and measures of statistical heterogeneity. If comparing groups, describe the direction of the effect. | - |
|  | 20c | Present results of all investigations of possible causes of heterogeneity among study results. | - |
|  | 20d | Present results of all sensitivity analyses conducted to assess the robustness of the synthesized results. | - |
| Reporting biases | 21 | Present assessments of risk of bias due to missing results (arising from reporting biases) for each synthesis assessed. | - |
| Certainty of evidence | 22 | Present assessments of certainty (or confidence) in the body of evidence for each outcome assessed. | - |
| **DISCUSSION** | | |  |
| Discussion | 23a | Provide a general interpretation of the results in the context of other evidence. | p. 13-14 |
|  | 23b | Discuss any limitations of the evidence included in the review. | p. 15 |
|  | 23c | Discuss any limitations of the review processes used. | p. 15 |
|  | 23d | Discuss implications of the results for practice, policy, and future research. | p. 13-15 |
| **OTHER INFORMATION** | | |  |
| Registration and protocol | 24a | Provide registration information for the review, including register name and registration number, or state that the review was not registered. | p. 4 |
|  | 24b | Indicate where the review protocol can be accessed, or state that a protocol was not prepared. | p. 4 |
|  | 24c | Describe and explain any amendments to information provided at registration or in the protocol. | - |
| Support | 25 | Describe sources of financial or non-financial support for the review, and the role of the funders or sponsors in the review. | p. 17 |
| Competing interests | 26 | Declare any competing interests of review authors. | p. 17 |
| Availability of data, code and other materials | 27 | Report which of the following are publicly available and where they can be found: template data collection forms; data extracted from included studies; data used for all analyses; analytic code; any other materials used in the review. | - |

**SUPPLEMENTAL TABLE 2: Full listed search**

**Search in PubMed, updated October 31, 2021**

(((((((breast neoplasm[MeSH Terms]) OR (((breast[MeSH Terms]) OR ((breast[Title/Abstract]) OR (mammary[Title/Abstract]))) AND ((Neoplasm[MeSH Terms]) OR (precancer*[Title/Abstract] OR cancer*[Title/Abstract] OR neoplas*[Title/Abstract] OR tumor[Title/Abstract] OR tumors*[Title/Abstract] OR tumour[Title/Abstract] OR tumours[Title/Abstract] OR carcinoma*[Title/Abstract] OR adenocarcinoma*[Title/Abstract] OR adeno carcinoma*[Title/Abstract] OR adenoma*[Title/Abstract] OR malignan*[Title/Abstract] OR lesion*[Title/Abstract])))) OR ((colorectal neoplasms[MeSH Terms]) OR (((((colon[MeSH Terms]) OR (Rectum[MeSH Terms])) OR (Anal canal[MeSH Terms])) OR (colon[Title/Abstract] OR colonic[Title/Abstract] OR bowel[Title/Abstract] OR rectal[Title/Abstract] OR rectum[Title/Abstract] OR sigmoid[Title/Abstract] OR anal[Title/Abstract])) AND ((Neoplasm[MeSH Terms]) OR (precancer*[Title/Abstract] OR cancer*[Title/Abstract] OR neoplas*[Title/Abstract] OR dysplas*[Title/Abstract] OR tumor[Title/Abstract] OR tumors*[Title/Abstract] OR tumour[Title/Abstract] OR tumours[Title/Abstract] OR carcinoma*[Title/Abstract] OR adenocarcinoma*[Title/Abstract] OR adeno carcinoma*[Title/Abstract] OR adenoma*[Title/Abstract] OR malignan*[Title/Abstract] OR lesion*[Title/Abstract]))))) OR ((((cervical intraepithelial neoplasia[MeSH Terms]) OR (uterine cervical neoplasm[MeSH Terms])) OR (uterine cervical dysplasia[MeSH Terms])) OR (((cervix uteri[MeSH Terms]) OR (cervix uteri[Title/Abstract] OR cervical[Title/Abstract] OR cervix[Title/Abstract] OR cervixes[Title/Abstract] OR cervices[Title/Abstract] OR cervico[Title/Abstract])) AND ((Neoplasms[MeSH Terms]) OR (precancer*[Title/Abstract] OR cancer*[Title/Abstract] OR neoplas*[Title/Abstract] OR dysplas*[Title/Abstract] OR dyskarios*[Title/Abstract] OR tumor[Title/Abstract] OR tumors*[Title/Abstract] OR tumour[Title/Abstract] OR tumours[Title/Abstract] OR carcinoma*[Title/Abstract] OR adenocarcinoma*[Title/Abstract] OR adeno carcinoma*[Title/Abstract] OR adenoma*[Title/Abstract] OR malignan*[Title/Abstract] OR lesion*[Title/Abstract] OR squamous[Title/Abstract] OR "small cell*"[Title/Abstract] OR "large cell*"[Title/Abstract]))))) AND (((((((Mass screening[MeSH Terms]) OR (population surveillance[MeSH Terms])) OR (Direct-to-consumer Screening and Testing[MeSH Terms])) OR (early diagnosis[MeSH Terms])) OR (secondary prevention[MeSH Terms])) OR (screening[Title/Abstract])) OR (early detection[Title/Abstract]))) AND (((((((((((((((((((((socioeconomic factors[MeSH Terms]) OR (Health Status Disparities[MeSH Terms])) OR (Healthcare Disparities[MeSH Terms])) OR (Health Equity[MeSH Terms])) OR (Health literacy[MeSH Terms])) OR (awareness[MeSH Terms])) OR (Minority Groups[MeSH Terms])) OR (Vulnerable Populations[MeSH Terms])) OR (population groups[MeSH Terms])) OR (urban population[MeSH Terms])) OR (Suburban Population[MeSH Terms])) OR (rural population[MeSH Terms])) OR (Patient Acceptance of Health Care[MeSH Terms])) OR (Physician Patient Relations[MeSH Terms])) OR (Health Knowledge, Attitudes, Practice[MeSH Terms])) OR (Persuasive Communication[MeSH Terms])) OR (health behavior[MeSH Terms])) OR (religion[MeSH Terms])) OR (Social Capital[MeSH Terms])) OR (Culture[MeSH Terms])) OR (socioeconomic inequalit*[Title/Abstract] OR socioeconomic inequit*[Title/Abstract] OR socioeconomic equalit*[Title/Abstract] OR socioeconomic equit*[Title/Abstract] OR health disparit*[Title/Abstract] OR health inequalit*[Title/Abstract] OR health inequit*[Title/Abstract] OR health equalit*[Title/Abstract] OR depriv*[Title/Abstract] OR underserved population*[Title/Abstract] OR gender[Title/Abstract] OR access[Title/Abstract] OR barrier*[Title/Abstract] OR obstacle*[Title/Abstract] OR challeng*[Title/Abstract] OR gap[Title/Abstract] OR gaps[Title/Abstract] OR facilitator*[Title/Abstract] OR religio*[Title/Abstract] OR social capital[Title/Abstract] OR culture*[Title/Abstract] OR ethni*[Title/Abstract] OR immigrant*[Title/Abstract]))) AND ((((((((((((((((((((Reminder Systems[MeSH Terms] ) OR (Online Systems[MeSH Terms])) OR (Health Information Systems[MeSH Terms])) OR (Medical Records[MeSH Terms])) OR (Physician Incentive Plans[MeSH Terms])) OR (Reimbursement, Incentive[MeSH Terms])) OR (Financial Support[MeSH Terms])) OR (Health Service[MeSH Terms])) OR (Delivery of Health Care[MeSH Terms])) OR (Guideline[MeSH Terms])) OR (Professional Practice[MeSH Terms])) OR (Education[MeSH Terms])) OR (Health Information Management[MeSH Terms])) OR (Information Dissemination[MeSH Terms])) OR (Feedback[MeSH Terms])) OR (Counseling[MeSH Terms])) OR (Community based participatory research[MeSH Terms])) OR (educat*[Title/Abstract] OR training[Title/Abstract] OR teach*[Title/Abstract] OR learn*[Title/Abstract] OR information*[Title/Abstract] OR remind*[Title/Abstract] OR system*[Title/Abstract] OR register*[Title/Abstract] OR recall*[Title/Abstract] OR assessment[Title/Abstract] OR incentiv*[Title/Abstract] OR support*[Title/Abstract] OR feedback[Title/Abstract] OR e-learn*[Title/Abstract] OR elearn*[Title/Abstract] OR online[Title/Abstract] OR course*[Title/Abstract])) AND ((Program Development[MeSH Terms]) OR (intervention*[Title/Abstract] OR experiment*[Title/Abstract] OR action*[Title/Abstract] OR implement* [Title/Abstract] OR trial [Title/Abstract]))) AND ((Health Personnel[MeSH Terms]) OR (provider*[Title/Abstract] OR practitioner*[Title/Abstract] OR personnel*[Title/Abstract] OR profession*[Title/Abstract] OR clinician*[Title/Abstract] OR health work*[Title/Abstract] OR general practition*[Title/Abstract] OR doctor*[Title/Abstract] OR physician*[Title/Abstract] OR nurs*[Title/Abstract])))) AND (((((((((Patient Participation[MeSH Terms]) OR (Community participation[MeSH Terms])) OR (Stakeholder Participation[MeSH Terms])) OR (Patient Compliance[MeSH Terms])) OR (Voluntary Programs[MeSH Terms])) OR (Health promotion[MeSH Terms])) OR (Program Evaluation[MeSH Terms])) OR (Outcome Assessment Health Care[MeSH Terms])) OR (participat*[Title/Abstract] OR uptake[Title/Abstract] OR adherence[Title/Abstract] OR coverage[Title/Abstract] OR [Title/Abstract] OR voluntary[Title/Abstract] OR attendance[Title/Abstract] OR utilization[Title/Abstract] OR utilisation[Title/Abstract] OR impact*[Title/Abstract] OR effect*[Title/Abstract] OR performance[Title/Abstract] OR examination[Title/Abstract] OR monitoring[Title/Abstract] OR rate*[Title/Abstract]))

**Search in Embase, updated on October 31, 2021**

| **#** | **Searches** | **Results** | **Annotations** |
| --- | --- | --- | --- |
| 1 | breast cancer/ or breast tumor/ | 470298 |  |
| 2 | breast/ | 90895 |  |
| 3 | (breast or mammary).ti,ab. | 694329 |  |
| 4 | neoplasm/ | 428981 |  |
| 5 | (precancer* or cancer* or neoplas* or tumor or tumors or tumour or tumours or carcinoma* or adenocarcinoma* or adeno carcinoma* or adenoma* or malignan* or lesion*).ti,ab. | 5534372 |  |
| 6 | 2 or 3 | 705823 |  |
| 7 | 4 or 5 | 5574287 |  |
| 8 | 6 and 7 | 584577 |  |
| 9 | 1 or 8 | 679108 | Breast cancer |
| 10 | rectum tumor/ | 13855 |  |
| 11 | colorectal carcinoma/ or colorectal tumor/ or colorectal cancer/ | 206136 |  |
| 12 | colon/ | 37265 |  |
| 13 | rectum/ | 35496 |  |
| 14 | anal canal/ | 6078 |  |
| 15 | (precancer* or cancer* or neoplas* or tumor or tumors or tumour or tumours or carcinoma* or adenocarcinoma* or adeno carcinoma* or adenoma* or malignan* or lesion).ti,ab. | 5067657 |  |
| 16 | (colon or colonic or bowel or rectal or rectum or sigmoid or anal).ti,ab. | 680058 |  |
| 17 | neoplasm/ | 428981 |  |
| 18 | 12 or 13 or 14 or 16 | 691201 |  |
| 19 | 15 or 17 | 5107938 |  |
| 20 | 18 and 19 | 319015 |  |
| 21 | 10 or 11 or 20 | 474951 | Colorectal cancer |
| 22 | "squamous intraepithelial lesion of the cervix"/ | 1121 |  |
| 23 | uterine cervix carcinoma in situ/ | 16371 |  |
| 24 | uterine cervix tumor/ | 16818 |  |
| 25 | uterine cervix dysplasia/ | 5261 |  |
| 26 | uterine cervix/ | 18581 |  |
| 27 | (precancer* or cancer* or neoplas* or dysplas* or dyskarios* or tumor or tumors or tumour or tumours or carcinoma* or adenocarcinoma* or adeno carcinoma* or adenoma* or malignan* or lesion* or squamous or "small cell*" or "large cell*").ti,ab. | 5616116 |  |
| 28 | (cervix uteri or cervical or cervix or cervixes or cervices* or cervico*).ti,ab. | 343911 |  |
| 29 | neoplasm/ | 428981 |  |
| 30 | 26 or 28 | 347291 |  |
| 31 | 27 or 29 | 5655986 |  |
| 32 | 30 and 31 | 180546 |  |
| 33 | 22 or 23 or 24 or 25 or 32 | 188109 | Cervical cancer |
| 34 | 9 or 21 or 33 | 1259565 | Breast OR colon OR cervical cancer |
| 35 | mass screening/ | 57845 |  |
| 36 | health survey/ | 205770 |  |
| 37 | early diagnosis/ | 115508 |  |
| 38 | secondary prevention/ | 30959 |  |
| 39 | Population Surveillance.ti,ab. | 380 |  |
| 40 | early detection.ti,ab. | 100254 |  |
| 41 | screening/ | 183208 |  |
| 42 | screening.ti,ab. | 813561 |  |
| 43 | 35 or 36 or 37 or 38 or 39 or 40 or 41 or 42 | 1243112 | Screening |
| 44 | socioeconomics/ | 148805 |  |
| 45 | health disparity/ | 24769 |  |
| 46 | health care disparity/ | 18544 |  |
| 47 | health equity/ | 5381 |  |
| 48 | health literacy/ | 14393 |  |
| 49 | awareness/ | 106042 |  |
| 50 | minority group/ | 16047 |  |
| 51 | vulnerable population/ | 21629 |  |
| 52 | population group/ | 3061 |  |
| 53 | urban population/ | 47904 |  |
| 54 | suburban population/ | 792 |  |
| 55 | rural population/ | 50317 |  |
| 56 | patient attitude/ | 72114 |  |
| 57 | doctor patient relationship/ | 5901 |  |
| 58 | attitude to health/ | 120689 |  |
| 59 | persuasive communication/ | 8986 |  |
| 60 | health behavior/ | 72009 |  |
| 61 | religion/ | 70871 |  |
| 62 | social capital/ | 3291 |  |
| 63 | (socioeconomic inequalit* or socioeconomic inequit* or socioeconomic equalit* or socioeconomic equit* or health inequalit* or health inequit* or health equalit* or depriv* or underserved population* or gender or access or barrier* or obstacle* or challeng* or gap or gaps or facilitator* or religio* or social capital or cultur* or ethni*).ti,ab. | 4411250 |  |
| 64 | immigrant*.ti,ab. | 30698 |  |
| 65 | 44 or 45 or 46 or 47 or 48 or 49 or 50 or 51 or 52 or 53 or 54 or 55 or 56 or 57 or 58 or 59 or 60 or 61 or 62 or 63 or 64 | 4901485 | Disadvantaged populations |
| 66 | 34 and 43 and 65 | 29526 | «P» in PICO |
| 67 | reminder system/ | 2843 |  |
| 68 | online system/ | 27972 |  |
| 69 | medical information system/ | 22035 |  |
| 70 | medical record/ | 189194 |  |
| 71 | personnel management/ | 58625 |  |
| 72 | reimbursement/ | 60725 |  |
| 73 | financial management/ | 116223 |  |
| 74 | health service/ | 166305 |  |
| 75 | health care delivery/ | 186934 |  |
| 76 | practice guideline/ | 475949 |  |
| 77 | professional practice/ | 56569 |  |
| 78 | education/ | 438690 |  |
| 79 | information dissemination/ | 22828 |  |
| 80 | feedback system/ | 84175 |  |
| 81 | counseling/ | 72628 |  |
| 82 | participatory research/ | 6178 |  |
| 83 | educat*.ti,ab. | 860947 |  |
| 84 | training.ti,ab. | 609888 |  |
| 85 | teach*.ti,ab. | 268724 |  |
| 86 | learn*.ti,ab. | 605194 |  |
| 87 | information*.ti,ab. | 1746108 |  |
| 88 | remind*.ti,ab. | 34015 |  |
| 89 | system*.ti,ab. | 4952121 |  |
| 90 | register*.ti,ab. | 306061 |  |
| 91 | recall*.ti,ab. | 100395 |  |
| 92 | assessment.ti,ab. | 1534196 |  |
| 93 | incentiv*.ti,ab. | 42140 |  |
| 94 | support*.ti,ab. | 2194019 |  |
| 95 | feedback.ti,ab. | 195332 |  |
| 96 | e-learn*.ti,ab. | 4838 |  |
| 97 | elearn*.ti,ab. | 714 |  |
| 98 | online.ti,ab. | 215348 |  |
| 99 | course*.ti,ab. | 893040 |  |
| 100 | 67 or 68 or 69 or 70 or 71 or 72 or 73 or 74 or 75 or 76 or 77 or 78 or 79 or 80 or 81 or 82 or 83 or 84 or 85 or 86 or 87 or 88 or 89 or 90 or 91 or 92 or 93 or 94 or 95 or 96 or 97 or 98 or 99 | 11712421 | Specific interventions |
| 101 | program development/ | 25172 |  |
| 102 | intervention*.ti,ab. | 1565149 |  |
| 103 | experiment*.ti,ab. | 2422676 |  |
| 104 | action*.ti,ab. | 1030999 |  |
| 105 | implement*.ti,ab. | 752485 |  |
| 106 | trial.ti,ab. | 964509 |  |
| 107 | 101 or 102 or 103 or 104 or 105 or 106 | 5995909 | Intervention |
| 108 | health care personnel/ | 189481 |  |
| 109 | provider*.ti,ab. | 273202 |  |
| 110 | practitioner*.ti,ab. | 203773 |  |
| 111 | personnel*.ti,ab. | 98633 |  |
| 112 | profession*.ti,ab. | 469242 |  |
| 113 | clinician*.ti,ab. | 363322 |  |
| 114 | health work*.ti,ab. | 28987 |  |
| 115 | general practition*.ti,ab. | 69343 |  |
| 116 | doctor*.ti,ab. | 193535 |  |
| 117 | physician*.ti,ab. | 590951 |  |
| 118 | nurs*.ti,ab. | 551954 |  |
| 119 | 108 or 109 or 110 or 111 or 112 or 113 or 114 or 115 or 116 or 117 or 118 | 2292044 | Providers |
| 120 | 100 and 107 and 119 | 459393 | «I» in PICO |
| 121 | patient participation/ | 30493 |  |
| 122 | community participation/ | 3207 |  |
| 123 | stakeholder engagement/ | 4306 |  |
| 124 | patient compliance/ | 140246 |  |
| 125 | voluntary program/ | 1466 |  |
| 126 | health promotion/ | 103077 |  |
| 127 | outcome assessment/ | 615844 |  |
| 128 | program evaluation/ | 17072 |  |
| 129 | performance/ | 34453 |  |
| 130 | examination/ | 94137 |  |
| 131 | (participat* or uptake or adherence or coverage or voluntary or attendance or utilization or utilisation or impact* or effect* or performance or examination or monitoring or rate*).ti,ab. | 15208273 |  |
| 132 | 121 or 122 or 123 or 124 or 125 or 126 or 127 or 128 or 129 or 130 or 131 | 15563346 | «O» in PICO |
| 133 | 66 and 120 and 132 | 2697 | P and I and O |
| **134** | **limit 133 to yr="2006 -Current"** | **2366** |  |

**Search in Web of Science, updated on October 31, 2021**

|  | **Query** | **Hits** | **Comments** |
| --- | --- | --- | --- |
| 1 | TI=(breast OR mammary) OR AB=(breast OR mammary) | 608,659 |  |
| 2 | TI=(precancer* OR cancer* OR neoplas* OR tumor OR tumors OR tumour OR tumours OR carcinoma* OR adenocarcinoma* OR “adeno carcinoma*” OR adenoma* OR malignan* OR lesion*) OR AB=(precancer* OR cancer* OR neoplas* OR tumor OR tumors OR tumour OR tumours OR carcinoma* OR adenocarcinoma* OR “adeno carcinoma*” OR adenoma* OR malignan* OR lesion*) | 4,330,102 |  |
| 3 | 1 AND 2 | 487,370 | Breast cancer |
| 4 | TI=(“cervical intraepithelial neoplas*” OR “uterine cervical neoplasm*” OR “uterine cervical dysplas*” OR «atypical squamous cells of the cervix" OR “squamous intraepithelial lesions of the cervix") OR AB=(“cervical intraepithelial neoplas*” OR “uterine cervical neoplasm*” OR “uterine cervical dysplas*” OR «atypical squamous cells of the cervix" OR “squamous intraepithelial lesions of the cervix") | 2,947 |  |
| 5 | TI=(“cervix uteri” OR cervical OR cervix OR cervixes OR cervices OR cervico*) OR AB=(“cervix uteri” OR cervical OR cervix OR cervixes OR cervices OR cervico*) | 238,453 |  |
| 6 | TI=(precancer* OR cancer* OR neoplas* OR dysplas* OR dyskarios* OR tumor OR tumors OR tumour OR tumours OR carcinoma* OR adenocarcinoma* OR “adeno carcinoma*” OR adenoma* OR malignan* OR lesion* OR squamous OR "small cell*" OR "large cell*") OR AB=(precancer* OR cancer* OR neoplas* OR dysplas* OR dyskarios* OR tumor OR tumors OR tumour OR tumours OR carcinoma* OR adenocarcinoma* OR “adeno carcinoma*” OR adenoma* OR malignan* OR lesion* OR squamous OR "small cell*" OR "large cell*") | 4,396,949 |  |
| 7 | 5 AND 6 | 121,974 |  |
| 8 | 4 OR 7 | 121,974 | Cervical cancer |
| 9 | TI=("colorectal neoplas*" OR "colonic neoplas*" OR "rectal neoplas*") OR AB=("colorectal neoplas*" OR "colonic neoplas*" OR "rectal neoplas*") | 5,934 |  |
| 10 | TI=(colon OR colonic OR bowel OR rectal OR rectum OR sigmoid OR anal OR anus) OR AB=(colon OR colonic OR bowel OR rectal OR rectum OR sigmoid OR anal OR anus) | 501,792 |  |
| 11 | TI=(precancer* OR cancer* OR neoplas* OR dysplas* OR tumor OR tumors OR tumour OR tumours OR carcinoma* OR adenocarcinoma* OR adeno carcinoma*OR adenoma* OR malignan* OR lesion*) OR AB=(precancer* OR cancer* OR neoplas* OR dysplas* OR tumor OR tumors OR tumour OR tumours OR carcinoma* OR adenocarcinoma* OR adeno carcinoma*OR adenoma* OR malignan* OR lesion*) | 4,351,650 |  |
| 12 | 10 AND 11 | 209,407 |  |
| 13 | 9 OR 12 | 212,513 | Colon cancer |
| 14 | 3 OR 8 OR 13 | 791,959 | All cancers |
| 15 | TI=("mass screening" OR "population surveillance" OR "early diagnosis" OR "secondary prevention" OR "early detection" OR screening) OR AB=("mass screening" OR "population surveillance" OR "early diagnosis" OR "secondary prevention" OR "early detection" OR screening) | 1,087,088 | Screening |
| 16 | TI=(“Socioeconomic Factor*” OR ”Health Disparit*” OR ”Health literacy” OR ”Awareness” OR ”Minority Group*” OR ”Vulnerable Population*” OR ”Population Group*” OR ”Urban Population*” OR ”Suburban Population*” OR ”Rural Population* OR  “Physician Patient Relation*” OR  ”Health Knowledge” OR “Health Attitude*” OR “Health practice” OR ”Persuasive Communication*” OR ”Health Behavior” OR ”socioeconomic inequalit*” OR “socioeconomic inequit*” OR “socioeconomic equalit*” OR socioeconomic equit*” OR “health disparit*” OR “health inequalit*” OR “health inequit*” OR “health equalit*” OR depriv* OR “underserved population*” OR gender OR access OR barrier* OR obstacle* OR challeng* OR gap OR gaps OR facilitator* OR religio* OR social capital OR culture* OR ethni* OR immigrant*) OR AB=(“Socioeconomic Factor*” OR ”Health Disparit*” OR ”Health literacy” OR ”Awareness” OR ”Minority Group*” OR ”Vulnerable Population*” OR ”Population Group*” OR ”Urban Population*” OR ”Suburban Population*” OR ”Rural Population* OR  “Physician Patient Relation*” OR  ”Health Knowledge” OR “Health Attitude*” OR “Health practice” OR ”Persuasive Communication*” OR ”Health Behavior” OR ”socioeconomic inequalit*” OR “socioeconomic inequit*” OR “socioeconomic equalit*” OR socioeconomic equit*” OR “health disparit*” OR “health inequalit*” OR “health inequit*” OR “health equalit*” OR depriv* OR “underserved population*” OR gender OR access OR barrier* OR obstacle* OR challeng* OR gap OR gaps OR facilitator* OR religio* OR social capital OR culture* OR ethni* OR immigrant) | 5,795,212 | Disadvantage populations |
| 17 | 14 AND 15 AND 16 | 12,911 | “P” in PICO |
| 18 | TI=(system* OR “Medical Record*” OR incentiv* OR reimbursement OR  support*OR “Health Service*” OR “Delivery of Health Care” OR “Guideline*” OR “Professional Practice” OR “Health Information Management” OR Counseling OR “Community based participatory research” OR educat* OR training OR teach* OR learn* OR information* OR remind* OR register* OR recall* OR assessment OR feedback OR e-learn* OR elearn* OR online OR course*) OR AB= (system* OR “Medical Record*” OR incentiv* OR reimbursement OR  support*OR “Health Service*” OR “Delivery of Health Care” OR “Guideline*” OR “Professional Practice” OR “Health Information Management” OR Counseling OR “Community based participatory research” OR educat* OR training OR teach* OR learn* OR information* OR remind* OR register* OR recall* OR assessment OR feedback OR e-learn* OR elearn* OR online OR course*) | 13,961,088 |  |
| 19 | TI=(“Program Development” OR intervention* OR experiment* OR action* OR implement* OR trial) OR AB=(“Program Development” OR intervention* OR experiment* OR action* OR implement* OR trial) | 9,080,927 |  |
| 20 | TI=(Personnel* OR provider* OR practitioner* OR profession* OR clinician* OR “health work*” OR “general practition*” OR doctor* OR physician* OR nurs*) OR AB=(Personnel* OR provider* OR practitioner* OR profession* OR clinician* OR “health work*” OR “general practition*” OR doctor* OR physician* OR nurs*) | 1,694,165 |  |
| 21 | 18 AND 19 AND 20 | 313,787 | “I” in PICO |
| 22 | TI=(participat* OR ”patient Compliance” OR voluntary OR  “Health promotion” OR ”Program Evaluation” OR ”Outcome Assessment” OR uptake OR adherence OR coverage OR attendance OR utilization OR utilization OR impact* OR effect* OR performance OR examination OR monitoring OR rate*) OR AB=(participat* OR ”patient Compliance” OR voluntary OR  “Health promotion” OR ”Program Evaluation” OR ”Outcome Assessment” OR uptake OR adherence OR coverage OR attendance OR utilization OR utilization OR impact* OR effect* OR performance OR examination OR monitoring OR rate*) | 20,523,693 | “O” in PICO |
| 23 | 17 AND 21 AND 22 | 933 | P and I and O |
| 24 | **Limit year 2006-current (October 31, 2021)** | **814** |  |

**SUPPLEMENT 3: PICO form used in PubMed**

| **P** | **I** | **O** |
| --- | --- | --- |
| Breast neoplasms  OR  ((Breast OR *breast*OR *mammary*) AND (Neoplasms OR *precancer* OR cancer* OR neoplas* OR tumor OR tumors* OR tumour OR tumours OR carcinoma* OR adenocarcinoma* OR adeno carcinoma* OR adenoma* OR malignan* OR lesion*))*  OR  Colorectal neoplasms  OR  ((Colon OR Rectum OR Anal canal OR*colon OR colonic OR bowel OR rectal OR rectum OR sigmoid OR anal*) AND *(N*eoplasms*OR precancer* OR cancer* OR neoplas* OR dysplas* OR tumor OR tumors* OR tumour OR tumours OR carcinoma* OR adenocarcinoma* OR adeno carcinoma*OR adenoma* OR malignan* OR lesion*))*  OR  Cervical intraepithelial neoplasia OR Uterine cervical neoplasms OR Uterine cervical dysplasia  OR  ((Cervix uteri *OR cervix uteri* OR *cervical OR cervix OR cervixes OR cervices OR cervico*)* AND (Neoplasms*OR precancer* OR cancer* OR neoplas* OR dysplas* OR dyskarios* OR tumor OR tumors* OR tumour OR tumours OR carcinoma* OR adenocarcinoma* OR adeno carcinoma* OR adenoma* OR malignan* OR lesion* OR squamous OR "small cell*" OR "large cell**")) | Reminder Systems OR  Online Systems OR  Health Information Systems OR  Medical Records OR  Physician Incentive Plans OR  Reimbursement, Incentive OR  Financial Support OR  Health Service OR  Delivery of Health Care OR  Guideline OR  Professional Practice OR  Education OR  Health Information Management OR  Information Dissemination OR  Feedback OR  Counseling OR  Community based participatory research  OR  *educat* OR training OR teach* OR learn* OR information* OR remind* OR system* OR register* OR recall* OR assessment OR incentiv* OR support * OR feedback OR e-learn* OR elearn* OR online OR course** | Patient Participation OR  Community participation OR  Stakeholder Participation OR  Patient Compliance OR  Voluntary Programs OR  Health promotion OR  Program Evaluation OR  Outcome Assessment Health Care  OR  *participat* OR uptake OR adherence OR coverage OR voluntary OR attendance OR utilization OR utilization OR impact OR effect* OR performance OR*  *examination OR monitoring OR rate** |
| AND    Mass screening OR Population Surveillance OR Direct-to-consumer Screening and Testing OR Early Diagnosis OR Secondary Prevention  OR  *Screening OR early detection* | AND    Program Development  *OR*  *intervention* OR experiment* OR action* OR implement* OR trial* |  |
| AND    Socioeconomic Factors OR Health Status Disparities OR Healthcare Disparities OR Health Equity OR Health Literacy OR Awareness OR Minority Groups OR Vulnerable Populations OR Population Groups OR Urban Population OR Suburban Population OR Rural Population OR Patient Acceptance of Health Care OR Physician Patient Relations OR Health Knowledge, Attitudes, Practice OR Persuasive Communication OR Health Behavior OR Religion OR Social Capital OR Culture  OR  *socioeconomic inequalit* OR socioeconomic inequit* OR socioeconomic equalit* OR socioeconomic equit* OR health disparit* OR health inequalit* OR health inequit* OR health equalit* OR depriv* OR underserved population* OR gender OR access OR barrier* OR obstacle* OR challeng* OR gap OR gaps OR facilitator* OR religio* OR social capital OR culture* OR ethni* OR immigrant** | AND    Health Personnel  *OR*  *provider* OR practitioner* OR personnel* OR profession* OR clinician* OR health work* OR general practition* OR doctor* OR physician* OR nurs** |  |

Table legend

PICO forms were used to structure the review question and the search strategy included following keywords [Title/Abstract] (in italic) and Mesh terms.
